# Resolution of inflammation and sepsis survival are improved by dietary Ω-3 fatty acids

**DOI:** 10.1038/cdd.2017.177

**Published:** 2017-10-20

**Authors:** Andreas Körner, Martin Schlegel, Julia Theurer, Hannes Frohnmeyer, Michael Adolph, Marieke Heijink, Martin Giera, Peter Rosenberger, Valbona Mirakaj

**Affiliations:** 1 Department of Anesthesiology and Intensive Care Medicine, University Hospital Tübingen, Eberhard-Karls University, Tübingen, Germany; 2 Center for Proteomics and Metabolomics, Leiden University Medical Center (LUMC), Leiden, The Netherlands

## Abstract

Critical conditions such as sepsis following infection or traumatic injury disturb the complex state of homeostasis that may lead to uncontrolled inflammation resulting in organ failure, shock and death. They are associated with endogenous mediators that control the onset of acute inflammatory response, but the central problem remains the complete resolution of inflammation. Omega-3 enriched lipid emulsions (Ω-3^+^ LEs) were used in experimental studies and clinical trials to establish homeostasis, yet with little understanding about their role on the resolution of inflammation and tissue regeneration. Here, we demonstrate that Ω-3 lipid emulsions (LEs) orchestrate inflammation-resolution/regeneration mechanism during sterile peritonitis and murine polymicrobial sepsis. Ω-3^+^ LEs recessed neutrophil infiltration, reduced pro-inflammatory mediators, reduced the classical monocyte and enhanced the non-classical monocytes/macrophages recruitment and finally increased the efferocytosis in sepsis. The actions of Ω-3^+^ LE were 5-lipoxygenase (5-LOX) and 12/15-lipoxygenase (12/15-LOX) dependent. Ω-3^+^ LEs shortened the resolution interval by 56%, stimulated the endogenous biosynthesis of resolution mediators lipoxin A4, protectin DX and maresin 1 and contributed to tissue regeneration. Ω-3^+^ LEs protected against hypothermia and weight loss and enhanced survival in murine polymicrobial sepsis. We highlighted a role of Ω-3^+^ LEs in regulating key mechanisms within the resolution terrain during murine sepsis. This might form the basis for a rational design of sepsis specific clinical nutrition.

The initiation and resolution of inflammation are complex processes characterized by the release of mediators that control the migration and the function of immune cells. This process is essential to exert successful protection against injury and/or infection. If particularly the resolution process fails, inflammation can become chronic leading to collateral tissue destruction and loss of functional organ integrity. Newly identified bioactive resolution phase lipid mediators such as arachidonic acid (AA)-derived lipoxins, eicosapentaenoic acid (EPA)-derived resolvins and docosahexaenoic derived resolvins, protectins (PDs) and maresins (MaRs) and their bioactive peptide-conjugate pathways are biosynthesized during the resolution phase. These so-called specialized pro-resolving lipid mediators (SPMs) actively stimulate cardinal signs of resolution, namely limitation of neutrophil influx, the counterregulation of pro-inflammatory mediators, apoptosis of PMN and the active clearance of apoptotic cells and invading microorganisms.^1^

Sepsis, a syndrome that is particularly marked by failed resolution of inflammation predisposes to metabolic and immunological dysfunction that causes high morbidity and mortality worldwide.^2, 3^ To date, however treatment for sepsis is nonspecific, focused primarily on symptomatic therapy. In recent years lipid emulsions have been tested in experimental and clinical trials in critically ill to evaluate a possible beneficial influence on inflammation. This showed a controversial beneficial role for Ω-3 supplementation in critically ill,^4, 5, 6^ meaning that so far, treatment strategies with reduced load of Ω-6 fatty acids such as fish oil-based, olive oil-based or medium-chain triglycerid-based LEs have not been recommended for critically ill because of the insufficient data.^7^ Discrepancies are still considered in the methodological bias including the optimum composition, dose and timeframes, and indication for parenteral LEs. In particular little information is available about the mechanism of LEs during the onset and the resolution of acute inflammation and the tissue regeneration.

In this report, we show that administration of Ω-3^+^ LEs control inflammation-resolution mechanisms. Using a self-limited acute inflammation model and a murine polymicrobial sepsis model we found dietary Ω-3^+^ LEs to stop neutrophil infiltration, to reduce pro-inflammatory cytokines and to enhance anti-inflammatory mediators. This was associated with a strong reduction of classical monocytes and an increase of non-classical monocyte/macrophage (MΦ) recruitment. Moreover, Ω-3^+^ LEs enhanced efferocytosis, whereas this phagocyte responses were lost in 12/15-LOX^−/−^ mice, suggesting that the actions of Ω-3^+^ LEs were 5-LOX and 12/15-LOX dependent. Ω-3^+^ LEs shortened the resolution interval, stimulated the local endogenous biosynthesis of SPMs and enhanced the tissue regeneration during peritonitis compared with vehicle control or the administration of Ω-3^−^ LEs. Moreover, Ω-3^+^ LEs protected against hypothermia and weight loss enhancing survival in murine polymicrobial sepsis. Together, these results demonstrate that Ω-3^+^ LEs control key innate protective mechanism during the onset and resolution of acute inflammation and promote to tissue repair and regeneration.

## Results

### Ω-3^+^ LE stimulates resolution of inflammation and promotes tissue regeneration

**Figure 1:**
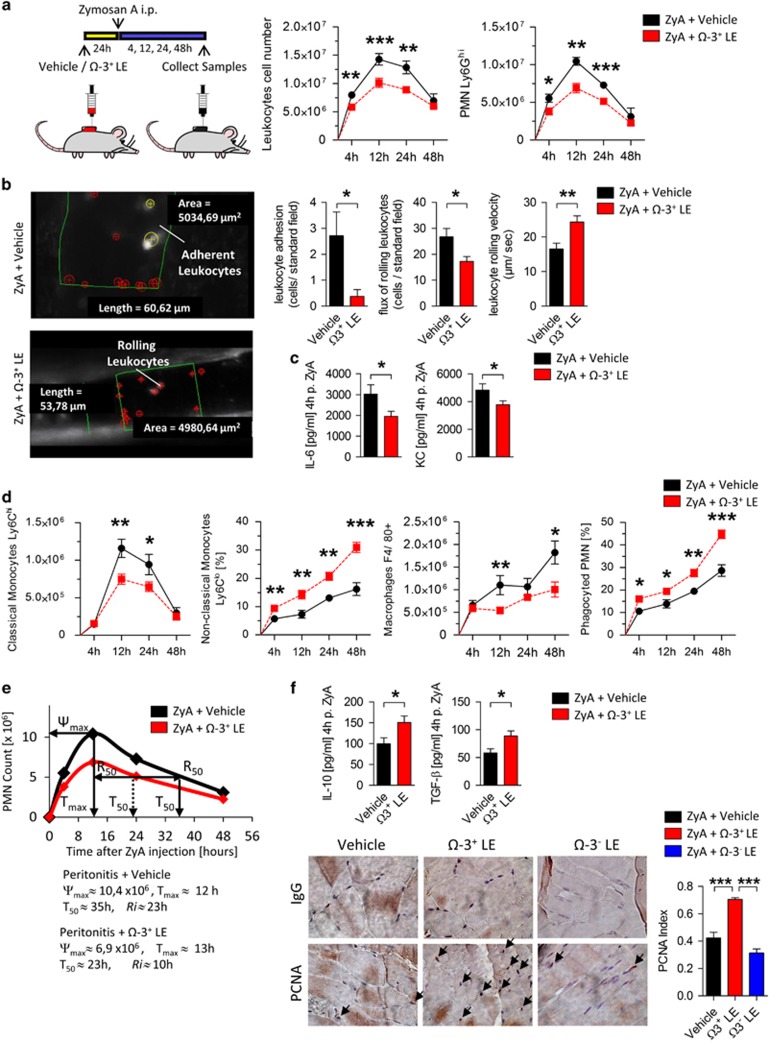
Ω-3^+^ LEs stimulate resolution of inflammation and promote tissue regeneration. (**a**) WT mice were exposed to Ω-3^+^ LE or vehicle for 24 h before injecting ZyA and then collecting peritoneal lavages at indicated time points. Total leukocytes were enumerated by light microscopy and neutrophils were determined by flow cytometry. (**b**) Leukocyte trafficking *in vivo* determined by intravital microscopy of postcapillary venules of the cremaster: Leukocyte adhesion, the flux of rolling leukocytes and rolling velocity after treatment with Ω-3^+^ LE or vehicle were quantified and represented by analog images. (**c**) Interleukin (IL)-6 and KC (IL-8 in humans) were determined in peritoneal lavages at 4 h. (**d**) Classical monocytes, non-classical monocytes, MΦ and efferocytosis were determined. (**e**) Resolution indices as previously defined:^37^ Ψ_max_ (maximal PMN counts), T_max_ (the time interval when PMN reach maximum), T_50_ (the time interval corresponding to 50% PMN reduction), R_i_ (the interval between Ψ_max_ and T_50_) (**f**) IL-10 and TGF-*β* levels were determined in peritoneal lavages at 4 h. Cell proliferation and regeneration at 24 h determined via immunohistochemical staining for PCNA in peritoneum and the calculated index. Results represent three independent experiments and are expressed as the mean±S.E.M., *n*=6–8 per group, *P<0.05; **P<0.01; ***P<0.001, two-tailed *t*-test vehicle *versus* Ω-3^+^ LE or one-way ANOVA, followed by Bonferroni’s multiple-comparison test

Given that inflammation and its timely resolution are held to be crucial for sufficient inflammatory responses that enable inflamed tissues to return to homeostasis we sought to determine the impact of Ω-3^+^ LEs, emulsions composed of long-chain, medium-chain fatty acids and fish oil (50:40:10) with Ω-6: Ω-3 ratio of 2.2:1 (Supplementary Table 1) on the dynamic of leukocytes. WT mice were pretreated with Ω-3^+^ LEs for 24 h prior injection of ZyA and lavages were collected at 4, 12, 24 and 48 h. Ω-3^+^ LE-treated mice displayed a drastic reduction in leukocyte infiltrates which was associated with a significant decrease of the PMN levels observed throughout the course of the inflammation when compared with vehicle control (Figure 1a). To directly corroborate the hypothesis that Ω-3^+^ LEs influence critical properties of neutrophils at the onset of acute inflammation, we sought to determine the impact of Ω-3^+^ LEs on the leukocyte-endothelium interactions by performing intravital microscopy of the murine cremaster. As shown in Figures 1b, Ω-3^+^ LEs significantly decreased the neutrophil adherence, the neutrophil migration and increased the rolling velocity in postcapillary venules. Representative microcirculation with and without Ω-3^+^ LE is shown in Supplementary Movies 2A and 2B. In these exudates, Ω-3^+^ LE also reduced IL-6 and keratinocyte chemoattractant (KC; IL-8 in humans; Figure 1c). Having demonstrated that Ω-3^+^ LEs impact the neutrophil recruitment in the early phase, we next focused on the resolution phase, where the recruitment of monocytes and MΦ predominate. The results showed that Ω-3^+^ LEs decreased the classical Ly6C^hi^ monocytes at the site of inflammation and increased the non-classical Ly6C^lo^ monocytes and MΦ that indicated a strong enhancement of MΦ clearance of apoptotic PMN (Figure 1d). To quantify the local kinetics of leukocyte infiltration, we determined the resolution indices (R*i*), demonstrating a 56% reduction in R*i* from 23 to 10 h in mice challenged with dietary Ω-3^+^ LE suggesting to strongly accelerate resolution of acute inflammation (Figure 1e). After having demonstrated that Ω-3^+^ LE displayed pro-resolving activity, we turned our attention to the possible impact on tissue repair and regeneration. Indeed, we found significantly increased exudate IL-10 and TGF-*β* levels that are known to be present in the resolution phase and to be an important factor on peritoneal healing (Figure 1f).^8, 9, 10^ To substantiate this hypothesis we performed immunohistochemical characterization of proliferating-cell-nuclear-antigen (PCNA), where Ω-3^+^ LE demonstrated a higher tissue regenerative response (Figure 1f). These results indicated that Ω-3^+^ LE might promote resolution mechanisms during peritonitis and improve tissue repair and regeneration.

### Ω-3^+^ LEs enhance pro-resolving lipid mediator biosynthesis

**Figure 2:**
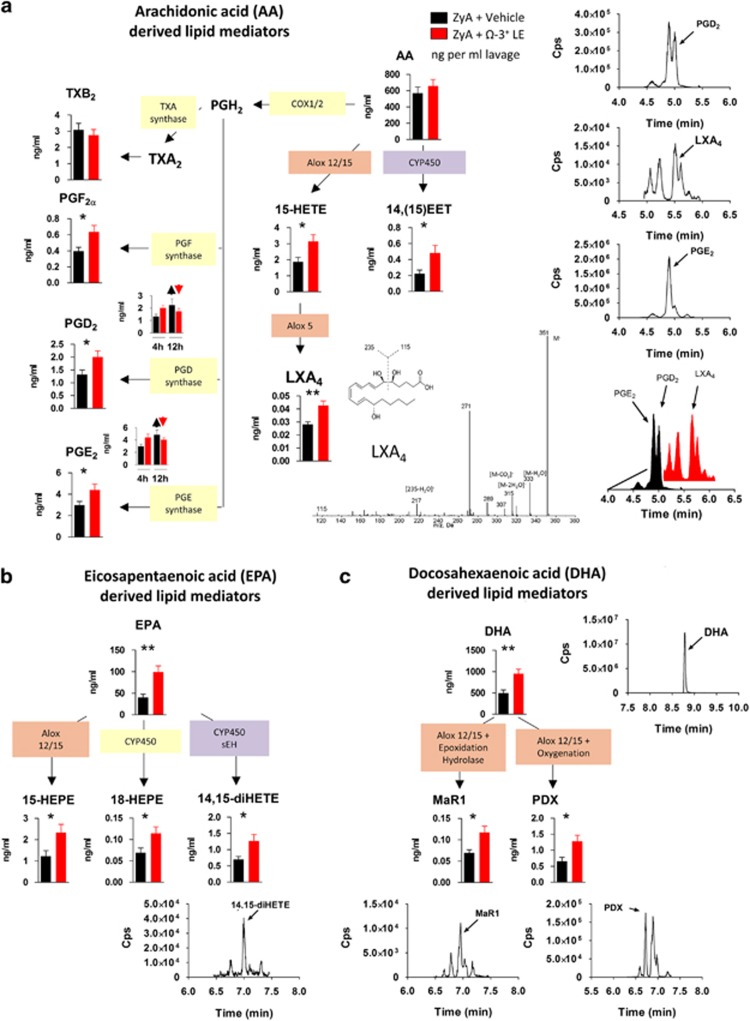
Ω-3^+^ LEs enhance pro-resolving lipid mediator biosynthesis. LC–MS/MS-based profiling was performed in peritoneal lavages of WT mice exposed to Ω-3^+^ LE or vehicle for 24 h before injecting ZyA. (**a**) Levels of bioactive lipid mediators and precursors derived from the arachidonic acid (AA) pathway with representative MS/MS spectra and MRM traces for the identified lipid mediators in peritoneal lavages at 4 h. (**b**) Levels of bioactive lipid mediators and precursors derived from the eicosapentaenoic acid (EPA) pathway and (**c**) the docosahexaenoic acid (DHA) pathway. Results represent three independent experiments and are expressed as the mean±S.E.M., *n*=6–8 per group, *P<0.05; **P<0.01; ***P<0.001, two-tailed *t*-test vehicle *versus* Ω-3+ LE

The resolution of acute inflammation is regulated by lipid mediator class-switching from production of pro-inflammatory lipid mediators in the initiation phase to the biosynthesis of SPM such as lipoxins, resolvins, protectins and maresins in the resolution phase. To explore whether Ω-3^+^ LEs impact the biosynthesis of these resolution phase mediators in murine peritonitis, we performed LC–MS/MS-based profiling of peritoneal lavages. In these, Ω-3^+^ LEs increased LXA_4_, MaR1 and PDX (Figures 2a–c). In addition Ω-3^+^ LEs significantly enhanced the arachidonic acid-derived product 15-hydroxyeicosatetraenoic acid (15-HETE), the eicosapentaenoic acid-derived products 15-hydroxyeicosapentaenoic acid (15-HEPE), 14, 15-dihydroxyeicosatetraenoic acid (14,15-diHETE) and 18-hydroxyeicosapentaenoic acid (18-HEPE) as well as the docosahexaenoic acid-derived product 17-HDHA. Ω-3^+^ LEs also increased 14,15-epoxyeiscosatrienoic acid (14,(15)-EET) that is known to possess anti-inflammatory and pro-resolving properties (Figure 2a).^11^ PGD_2_ and PGE_2_ levels were markedly enhanced at 4 h following administration of Ω-3^+^ LEs compared with mice challenged with ZyA alone, whereas at 12 h both factors were decreased indicating that Ω-3^+^ LEs induced a mediator class switch from prostaglandins to the biosynthesis of anti-inflammatory and pro-resolving mediators (Figure 2a; Supplementary Figure 1; Supplementary Table 2). Taken together, these results indicate that Ω-3^+^ LEs altered the LM profile in murine peritonitis toward a pro-resolving LM-SPM signature profile with pro-resolving characteristics and as such enhances tissue regeneration.

### Ω-3^−^ LEs display impaired pro-resolving properties

**Figure 3:**
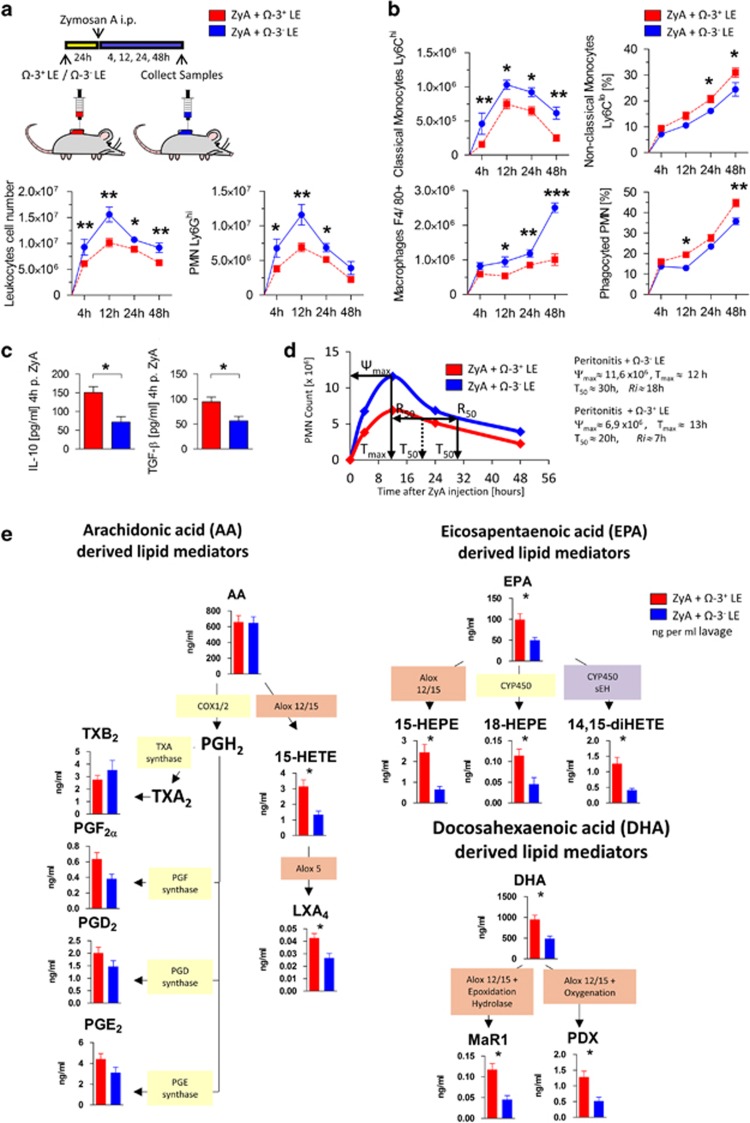
Ω-3^−^ LEs diminish pro-resolving properties during inflammation. WT mice were exposed to Ω-3^−^ LE or Ω-3^+^ LE for 24 h before injecting ZyA and then collecting peritoneal lavages. (**a**) Total leukocytes were enumerated by light microscopy and neutrophils were determined by flow cytometry. (**b**) Classical monocytes, non-classical monocytes, MΦ and efferocytosis of apoptotic PMNs were determined by flow cytometry. (**c**) IL-10 and TGF-*β* levels in peritoneal lavages at 4 h. (**d**) Resolution indices.^37^ (**e**) Levels of endogenous pro-resolving mediators assessed by LC–MS/MS-based analysis. Results represent three independent experiments with *n*=6-8 mice and are expressed as the mean±S.E.M., *n*=6–8 per group, *P<0.05; **P<0.01; ***P<0.001, two-tailed *t* -test Ω-3+ LE versus Ω-3- LE

To reflect the clinical routine and evaluate generally used nutrition solutions we further compared the impact of Ω-3^+^ LEs with non-enriched Ω-3 (Ω-3^−^) LEs (emulsions composed of long-chain and medium-chain fatty acids (50:50) with Ω-6: Ω-3 ratio of 6.6:1) (Supplementary Table 1) on the resolution programs in murine peritonitis. Mice treated with Ω-3^−^ LEs displayed a lower impact on the resolution mechanism as assessed by increased infiltration of PMN (Figure 3a) and classical monocytes accompanied by decreased non-classical monocytes that indicated a strong reduction of MΦ clearance of apoptotic PMN compared with Ω-3^+^ LEs treated mice (Figure 3b). To further validate the opposing impact of Ω-3^−^ LEs on the resolution of acute inflammation, we determined the exudate IL-10 and TGF-*β* levels and the characterization of tissue PCNA that contribute to resolution and regenerative programs^8^ (Figures 3c and 1f). Here, we found a significant reduction of both cytokines and impaired tissue regenerative response following Ω-3^−^ LEs administration compared with Ω-3^+^ LEs treated mice (Figure 1f). When exploring the resolution index, we found an increase in R*i* from 7 to 18 h in Ω-3^−^ LEs treated mice (Figure 3d). When exploring the LM profiles we found significantly lower exudate levels of LXA_4_, MaR1, PDX, 15-HETE 15-HEPE and 18-HEPE in Ω-3^−^ LEs treated mice (Figure 3e). Together, these data reflect impaired resolution of inflammation and tissue repair in murine peritonitis when treated with Ω-3^−^ LEs as assessed by increased recruitment of PMN and classical monocytes, reduced non-classical monocytes and efferocytosis, reduced SPM biosynthesis, elongation of the resolution interval and reduced expression of PCNA compared with Ω-3^+^ LE-treated peritonitis mice.

### Ω-3^+^ LEs enhance human MΦ function, efferocytosis and phagocytosis

**Figure 4:**
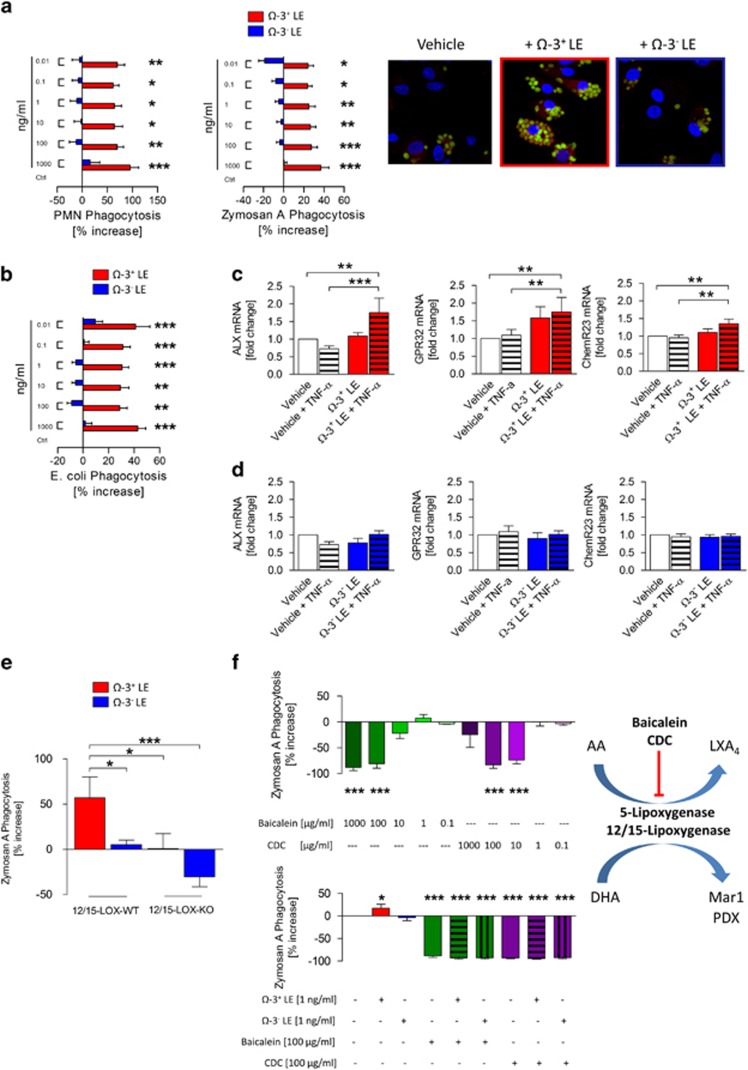
Ω-3^+^ LEs enhance human MΦ efferocytosis and phagocytosis through 5-LOX and 12/15-LOX. Human MΦ were incubated with indicated concentrations of Ω-3^+^ or Ω-3^−^ LEs. (**a**) The rate of efferocytosis of apoptotic PMN or ZyA-labeled particles was assessed by immunofluorescence and illustrated by immunofluorescence images. (**b**) The rate of efferocytosis of fluorescently labeled *E. coli* bacteria was performed using a fluorescent reader. (**c**) mRNA expression of ALX/FPR2, DRV1/GPR32 and ERV/ChemR23 on human MΦ following stimulation with Ω-3^+^ LEs or (**d**) Ω-3^−^ LEs. The results are representative of 8–14 independent experiments and are expressed as the mean±S.E.M., *P<0.05; **P<0.01; ***P<0.001, one-way ANOVA, followed by Dunnett’s multiple-comparison test. In a different experiment, (**e**) peritoneal MΦ from WT or 12/15-LOX^−/−^ mice were incubated with Ω-3^+^ or Ω-3^−^ LEs, and the rate of phagocytosis of fluorescently labeled ZyA particles was assessed using a fluorescent plate reader. (**f**) Human MΦ were incubated with indicated concentrations of baicalein and CDC and the degree of phagocyted fluorescently labeled ZyA particles was assessed. Human MΦ were incubated with Ω-3^+^ or Ω-3^−^ LEs in the absence or presence of baicalein or CDC and the rate of MΦ clearance of ZyA particles was performed. Results are representative of 5–10 independent experiments and are expressed as the mean±S.E.M., *P<0.05; **P<0.01; ***P<0.001, one-way ANOVA, followed by Dunnett’s or Bonferroni’s multiple-comparison test

As mentioned above, of great importance for promoting resolution of inflammation is the successful clearance of pathogens and inflammatory cells. Having demonstrated pro-resolving properties of Ω-3^+^ LEs in murine peritonitis, for human translation, we explored the ability of Ω-3^+^ and Ω-3^−^ LEs to firstly promote human MΦ efferocytosis of apoptotic PMN and phagocytosis of ZyA particles (Figure 4a) and secondary the MΦ phagocytosis of *Escherichia coli* as a feature for the infection-resolving actions (Figure 4b). Consistent with the *in vivo* findings, Ω-3^+^ LEs significantly increased the capacity of primary human MΦ to uptake apoptotic human PMNs, ZyA particles and *E. coli* bacteria. Of interest, we also found these results not to be affected through Ω-3^−^ LEs (Figures 4a and b). Because GPCR receptors such as ALX/FPR2, DRV1/GPR32 and ERV/ChemR23 have been demonstrated to mediate pro-resolving actions at low concentrations,^12^ we next determined the expression of these receptors on human MΦ following stimulation with vehicle or TNF-α or Ω-3^+^ LEs±TNF-*α* for 4 h. As expected, we found increased mRNA levels of ALX/FPR2, DRV1/GPR32 and ERV/ChemR23 when treated with Ω-3^+^ LEs+TNF-*α* compared with the control group (Figure 4c). Importantly, MΦ stimulated with Ω-3^−^ LEs failed to increase the expression of these GPCR receptors (Figure 4d). Taken together these data support the role of Ω-3^+^ LEs as activator of pro-resolving mechanisms.

### The actions of Ω-3^+^ LE are 5-LOX and 12/15-LOX dependent

Because the enzyme 12/15-lipoxygenase was identified to contribute to the generation of pro-resolving mediators, we next sought to investigate whether Ω-3^+^ LEs affect the phagocytosis in 12/15-lipoxygenase KO (12/15-LOX^−/−^) MΦ. Peritoneal MΦ from WT and 12/15-LOX^−/−^ mice were incubated with Ω-3^+^ LEs or Ω-3^−^ LEs. The impact of Ω-3^+^ LEs on MΦ phagocytosis was significantly reduced in 12/15-LOX^−/−^ mice, suggesting that the action of Ω-3^+^ LEs is 12/15-LOX dependent (Figure 4e). Similar actions were observed with human MΦ where Ω-3^+^ LEs did not display phagocytic impact in MΦ stimulated with 5-lipoxygenase and 12/15-lipoxygenase inhibitors baicalein^13^ or cinnamyl-3,4dihydroxy-α-cyanocinnamate (CDC; Figure 4f).

### Ω-3^+^ LEs improve survival in murine sepsis

**Figure 5:**
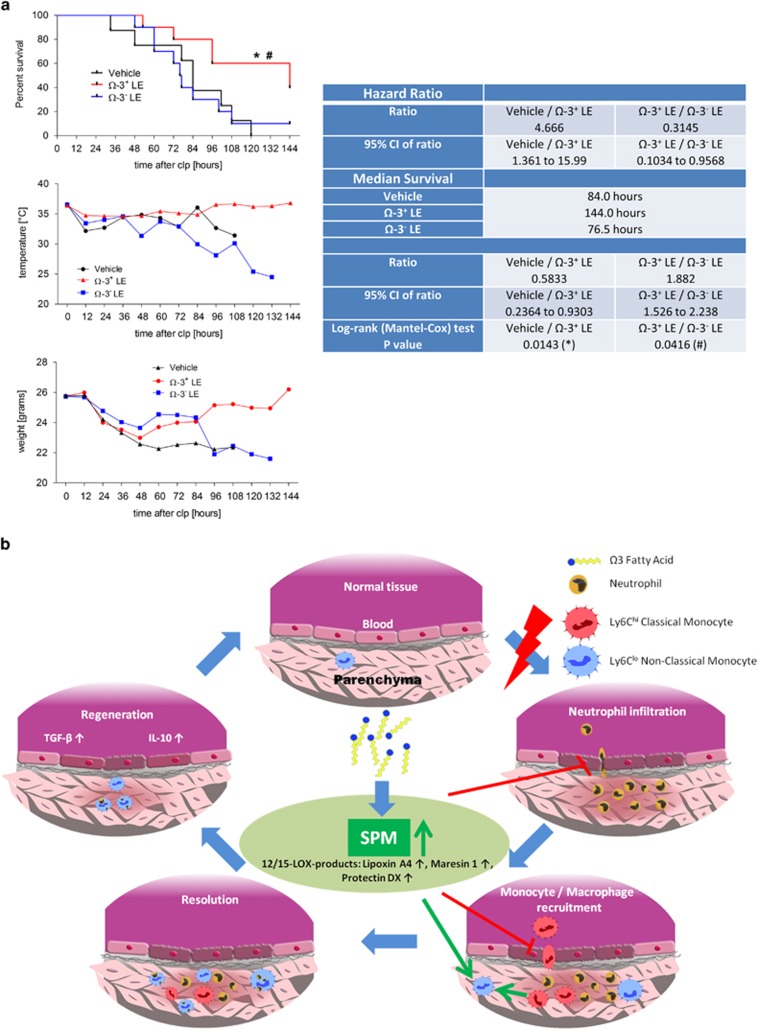
Ω-3^+^ LEs improve survival in murine sepsis. (**a**) WT animals were administered with Ω-3^+^ LEs or vehicle 24 h before CLP and survival, body temperature and body weight assessed over 6 days. (**b**) Schematic representation of the flow of information contributing to the impact of Ω-3^+^ LEs on the initiation and the resolution of acute inflammation and the tissue regeneration. Results represent two independent experiments with *n*=6–8 mice and are expressed as the mean (temperature and weight) *n*=6–8 per group, log-rank (Mantel–Cox) test, *P<0.05 vehicle *versus* Ω-3^+^ LE, ^#^*P*<0.05 Ω-3^+^ LE *versus* Ω-3^−^ LE

To investigate whether the observed beneficial effects of Ω-3^+^ LEs could decrease mortality owing to polymicrobial sepsis we performed a survival test in cecal ligation and puncture (CLP) model. Figure 5 shows that the administration of Ω-3^+^ LEs reduced the mortality rates and increased survival up to 60%, respectively. Since hypothermia is a risk factor for increased mortality in ICU patients with infection, we determined the body (surface) temperature and the weight of the infected mice treated with Ω-3^+^ LEs or Ω-3^−^ LEs (Supplementary Movies 1A–F are shown in the Supplementary Methods). Notably, Ω-3^+^ LEs protected mice from hypothermia and weight loss compared with the vehicle group (Figure 5a). By contrast, mice that were treated with Ω-3^-^ LEs did neither improve the survival nor protect from hypothermia and weight loss (Figure 5a). To corroborate that this improved outcome was due to the production of SPMs we carried out additional experiments to determine the impact of Ω-3^+^ LEs in the CLP model. For this purpose, C57BL/6 mice were administered with Ω-3^+^ LEs, Ω-3^−^ LEs or vehicle 24 h prior exposure to CLP and lavages were collected at 4 h. Collected results showed that mice treated with Ω-3^+^ LEs demonstrated a significant reduction in leukocyte infiltrates that was combined with a significant reduction of PMN when compared with Ω-3^−^ LEs or vehicle (Supplementary Figure 4A). Moreover, the results showed increased non-classical monocyte levels that indicated a strong enhancement of the MΦ efferocytosis of apoptotic PMN (Supplementary Figure 4B). Next, we determined the impact of Ω-3^+^ LEs and Ω-3^−^ LEs on the biosynthesis of the lipid resolution phase mediators. In the peritoneal lavages Ω-3^+^ LEs increased significantly the specialized pro-resolving mediators LXA_4_, MaR1 and PDX and the arachidonic acid-derived product 15-hydroxyeicosatetraenoic acid (15-HETE) compared with Ω-3^−^ LEs or vehicle (Supplementary Figure 5). In contrast, Ω-3^+^ LEs significantly reduced the pro-inflammatory LTB_4_. Taken together, these data indicate that Ω-3^+^ LEs also demonstrated anti-inflammatory and pro-resolving effects during peritoneal infection when compared with Ω-3^−^ LEs or vehicle suggesting that this improved outcome was due to the biosynthesis of SPMs.

## Discussion

In the present study, we report that dietary Ω-3 LEs significantly controls inflammation-resolution mechanisms. Using a peritonitis and a sepsis model we found Ω-3^+^ LEs to accelerate resolution of inflammation, shortening the R_i_ from 23 to 10 h. Ω-3^+^ LE stopped neutrophil infiltration, reduced pro-inflammatory and enhanced anti-inflammatory mediators. Also, Ω-3^+^ LEs strongly reduced the classical monocytes and increased the non-classical monocyte/ MΦ recruitment and finally enhanced efferocytosis of apoptotic PMN. These phagocyte responses were lost in 12/15-LOX^−/−^mice, suggesting that the actions of Ω-3^+^ LEs were 12/15-LOX dependent. Ω-3^+^ LEs stimulated the local endogenous biosynthesis of SPMs that have been demonstrated to actively enhance resolution of inflammation and tissue regeneration compared with peritonitis alone or peritonitis and Ω-3^−^ LE treatment. Moreover administration of Ω-3^+^ LEs protected against hypothermia and weight loss and enhanced survival in murine sepsis. Together, these results show that Ω-3^+^ LEs control key innate protective mechanism during the onset and the resolution of acute inflammation and promote survival in sepsis.

Although infection frequently underlies sepsis this is not entirely the case, more than 40% of cases are caused by sterile/non-infective processes.^2^ Unresolved immunological processes are one of the key causes that lead to persistent critical illness during sepsis and the development of organ dysfunction. Despite improved management concepts for sepsis, the mortality with no targeted treatment remains still high. The complex pathophysiology of sepsis is marked by two phases, the inflammatory storm where host- and pathogen-derived classical signals interact dangerously with each other and the anti-inflammatory phase.^14^ The anti-inflammatory response is characterized by the interplay between humoral, cellular and neuronal mechanisms that potentially mitigate the detrimental effects of the pro-inflammatory response. Particularly, innate cells such as monocytes and MΦ change to an anti-inflammatory phenotype that activates resolution and regeneration programs. Efficient resolution of inflammation is an active process activating endogenous mechanisms to promote a return to tissue homeostasis.^1^ Newly identified a novel genus of bioactive LMs – namely lipoxins, resolvins, protectins and maresins – known as SPMs possess anti-inflammatory and pro-resolving capacity.^1, 15, 16^ During the early onset phase and the resolution phase these SPMs biosynthesized from essential fatty acids are produced locally and exert protective actions on leukocytes, activate efferocytosis, promote tissue regeneration and reduce pain.^1, 17, 18^ PGE_2_ and PGD_2_ in addition to their roles in the initiation of an inflammatory response may undergo a temporal mediator class switch to produce pro-resolving mediator such us a lipoxins and SPM, indicating that the beginning signals the termination of the acute inflammatory response.^19^ In this context, reduced dietary intake of Ω-3 (EPA and DHA) could reduce the biosynthesis of SPMs contributing to failed resolution and disease pathologies.

Over the last decades, diverse strategies for nutrition therapies with LEs have been determined in various experimental studies and clinical trials.^20^ It is generally recognized that intravenous LEs composed of predominantly long-chain polyunsaturated fatty acid (e.g., soybean oil) may negatively influence inflammatory processes of critically ill.^21, 22^ Following the concerns that have been raised with respect to the *in vitro*, *in vivo* and clinical studies the generation of alternative intravenous LEs containing medium-chain tricglycerides, fish oil and olive oil with or without addition of soybean oil have been developed.^23^ Recently, in a secondary analysis of data from four International Nutrition Surveys the effects of different classes of lipid emulsions on clinical outcomes in critically ill were examined.^24^ The main findings and conclusion of this study demonstrated an association of fish oil or olive oil-based LEs with improvement in clinical outcomes and mechanical ventilation compared with soybean oil-based LEs.^25^ Interestingly, however, no overall impact on infections was shown. In a further meta-analysis, Pradelli *et al.* reported fish oil containing LEs to reduce infections in elective surgical and ICU patients and to decrease the length of stay, both in the ICU and in hospital overall. In this meta-analysis no statistically significant effect on mortality was found.^26^ Subsequently, contradictory and inconclusive results were demonstrated in systematic reviews of studies and subgroup analysis.^27, 28, 29^ Nevertheless, because the finding of the clinical trials and experimental reports are still inconsistent in demonstrating clinical benefits in the ICU, the current guidelines do not make a recommendation on the types of lipids to be used in critically ill.^20^ Disagreements are still considered in the methodological bias including the optimum composition, dose and timeframes, and indication for parenteral LEs.

On the basis of the factual situation and since the current focus in research has moved from inhibiting inflammation to accelerating resolution of inflammation, we intended to determine the impact of Ω-3^+^ and Ω-3^−^ LEs on the biochemical mechanism during the onset and the resolution of acute inflammation. In murine sepsis we found that Ω-3^+^ LEs influenced the dynamic of leukocytes where it reduced the neutrophil infiltration throughout the course of inflammation by particularly decreasing the adherence and the neutrophil migration and increasing the rolling velocity of the PMNs in the early phase of inflammation. Ω-3^+^ LEs decreased the classical Ly6C^hi^ monocytes at the site of inflammation and increased the non-classical Ly6C^lo^ monocytes and the MΦ that indicated a strong enhancement of MΦ clearance of apoptotic PMN. The R*i* was reduced by 56% in mice treated with Ω-3^+^ LEs compared with the vehicle control. Since pro-resolution is a distinguishing procedure from anti-inflammation where agonists of resolution such as SPM play a crucial role in the non-phlogistic clearance from sites of inflammation, we found Ω-3^+^ LEs to significantly increase levels of LXA_4_, MaR1 and PDX in both, the sterile peritonitis and murine microbial sepsis. In addition Ω-3^+^ LEs enhanced the arachidonic acid-derived product 15-HETE, the eicosapentaenoic acid-derived products 15-HEPE, 14,15-diHETE and 18-HEPE as well as the docosahexaenoic acid-derived product 17-HDHA. Ω-3^+^ LEs also increased 14,(15)EET that is known to possess anti-inflammatory and pro-resolving properties. MΦ have a crucial role in wound healing and organ regeneration. In wound healing inflammatory monocytes accumulate in the injured tissue and particularly phagocytosis of tissue debris can induce mononuclear cells to switch from pro-inflammatory to an anti-inflammatory phenotype. It is well known that M2 cells – monocytes and/or MΦ – express high levels of anti-inflammatory mediators such as IL-10 and TGF-*β*^10^ that contribute to the rapid resolution and wound healing through (e.g.) recruiting fibroblasts into the wound site to promote myofibroblast differentiation.^30, 31, 32^ Our data demonstrate high levels of IL-10 and TGF-*β* following Ω-3^+^ supplementation compared with vehicle control or Ω-3^−^ LE, suggesting a positive influence in the peritoneal healing. To substantiate these data we performed immunohistochemical characterization of PCNA where omega-3 supplementation demonstrated higher tissue regenerative response. To further explore the impact of Ω-3^+^ LE also in the presence of an underlying infection process, we used a murine CLP sepsis model, demonstrating protection against hypothermia and weight loss and enhancement in survival. Of note, phagocyte responses were lost in 12/15-LOX^−/−^ mice, indicating that the actions of Ω-3^+^ LEs were 12/15-LOX dependent. Conversely, the administration of Ω-3^−^ LEs, did neither improve the resolution nor the survival during sepsis, suggesting that the Ω-3^+^ component of the dietary LEs is crucial to impact the criteria of resolution of inflammation.

Hence, the present results implicate a critical role for Ω-3^+^ LEs in modulating inflammation, infection and stimulating mechanisms of resolution and tissue regeneration and provides novel evidence for the performance of clinical investigations in the future.

## Materials and methods

Methods and any associated references are available in the Supplementary Information.

### Animals

The Institutional Review Board and the Regierungspräsidium Tübingen approved this project. 12/15-LOX-deficient mice (12/15-LOX^−/−^) and littermate control mice were bred and genotyped as previously described.^33^

### Peritonitis

Zymosan A (ZyA, Invivogen, San Diego, CA, USA) was prepared in a 1 mg/ml solution in saline and 1 mg was injected i.p. After 4, 12, 24 and 48 h, C57BL/6 mice were euthanized with pentobarbital (100 mg/kg body weight) and peritoneal lavage was performed using ice-cold PBS (without Calcium or Magnesium). Peritoneal cavity was gently massaged and lavage was withdrawn. Subsequently, organs were harvested for further analysis.

### Intravital microscopy of cremaster microvasculature

ZyA induced peritonitis was implemented as described above. Rhodamine-6G (Sigma-Aldrich, St. Louis, MO, USA) was injected i.v. to stain circulating leukocytes. The cremaster microcirculation was observed *in vivo* using a Nikon Eclipse Ci-L microscope (Nikon, Düsseldorf, Germany).

### Cecal ligation and puncture

Cecal ligation and puncture (CLP) procedure in C57BL/6 mice was performed as described previously.^34^ Following the induction of anesthesia a longitudinal skin midline incision is done and linea alba should be identified and dissected to gain access to peritoneal cavity. Cecum is located and exterioized by blunt forceps to prevent damage of the mesenterial blood vessels and intestine and is ligated 50%, which correlates with a mid-grade sepsis. Subsequently the distal part of the cecum is perforated with a 20-Gauge needle by through-and-through puncture. The cecum is relocated to peritoneal cavity and peritoneum and skin is closed with 5-0 sutures and the animals are resuscitated with 1 ml of prewarmed saline.

### Differential leukocyte counts, facs analysis and cytokines

Exudate cells from the peritoneal lavages were prepared to determine the leukocyte subtypes. The cells were blocked with anti-mouse CD16/CD32 and stained with anti-mouse APC-Ly6G, e450-F4/80 (all from eBioscience, San Diego, CA, USA) and FITC-Ly6C (BioLegend, San Diego, CA, USA) for 30 min at 4 °C. To determine the efferocytosis rate *in vivo,* cells were permeabilized, then stained with PerCP-Cy5.5-conjugated anti-Ly6G (eBioscience) and analyzed using flow cytometry (BD FACSCanto II) (Supplementary Figure 2). Cytokines were measured in the murine peritoneal exudates using standard ELISA (R&D Systems, Minneapolis, MN, USA).

### Human MΦ efferocytosis and phagocytosis

Carboxyfluorescein diacetate labeled human PMNs were allowed to undergo apoptosis in serum-free medium for 16–18 h. MΦ were then incubated with Ω-3^+^ (Lipidem, B.Braun) or Ω-3^−^ (Lipofundin, B.Braun) LEs or vehicle. Apoptotic PMNs were added at a 1:3 ratio (MΦ:PMN) and incubated for 60 min to induce phagocytosis. In separate experiments, MΦ were preincubated either with Ω-3^+^ or Ω-3^−^ LE or vehicle and then incubated with ZyA particles (Molecular Probes, Eugene, OR, USA) at a 1:30 ratio (MΦ:ZyA particles) or *E. coli* particles at a 1:50 ratio (MΦ:*E. coli*) for 60 min. In a further experiment, human MΦ were incubated with baicalein (Santa Cruz Biotechnology) or cinnamyl-3,4dihydroxy-α-cyanocinnamate (CDC, Santa Cruz Biotechnology) and the degree of phagocytosis was assessed by using a fluorescent plate reader (Tecan, Männedorf, Switzerland).

### Transcriptional analysis of SPM receptors

Transcriptional analysis was performed using the following primers: *GPR32:* 5′-TGG ACC GTT GCA TCT CTG TC-3′, 5′- AGT GCG TAC AGC CAT TCC AT-3′ C*hemR23*: 5′-AGG GAC TGA TTG GCT GAG GA-3′, 5′-ATC CTC CAT TCT CAT TCA CCG T-3′ *ALX:* 5′-TGT TCT GCG GAT CCT CCC ATT-3′, 5′-CTC CCA TGG CCA TGG AGA CA -3′. 18S- expression was evaluated with sense 5′-GTA ACC CGT TGA ACC CCA TT-3′ and antisense 5′-CCA TCC AAT CGG TAG TAG CG-3′.

### LC–MS/MS

The targeted lipidomics and lipid mediator studies were performed by MG and MH in the Center for Proteomics and Metabolomics, Leiden University Medical Center (LUMC), The Netherlands. Peritoneal lavages were thawed and internal standards added. The samples were extracted twice using MeOH and prepared for analysis according to published protocols.^35^ LC–MS/MS analysis was carried out using a 6500 QTrap LC–MS/MS system as described in Heemskerk *et al.*^36^

### Statistics

All data are presented as mean±S.E.M. Statistical analysis was performed with GraphPad 5.0 software (GraphPad, San Diego, CA, USA). Two-tailed Student’s *t*-test or one-way ANOVA, followed by Bonferroni’s or Dunnett’s multiple-comparison test were applied as appropriate considering *P*-values <0.05 significant.
