# Supplementary material for: Resolution of inflammation and sepsis survival are improved by dietary Ω-3 fatty acids
Source: Cell Death Differ. 2017 Oct 20;25(2):421–31. doi: 10.1038/cdd.2017.177 (PMC5762854; doi:10.1038/cdd.2017.177)
Supplement: Supplementary Information [file cdd2017177x1.docx]

**Supplementary Information**

**Resolution of Inflammation and Sepsis Survival Are Improved by Dietary Ω-3 Fatty Acids**

Andreas Körner^1^, Martin Schlegel^1^, Julia Theurer^1^, Hannes Frohnmeyer^1^, Michael Adolph^1^, Marieke Heijink^2^, Martin Giera^2^, Peter Rosenberger^1^ and Valbona Mirakaj^1^

^1^Department of Anesthesiology and Intensive Care Medicine, University Hospital Tübingen,

Eberhard-Karls University Tübingen, Germany

^2^Center for Proteomics and Metabolomics, Leiden University Medical Center (LUMC), The Netherlands

**METHODS**

**Implantation of Infusion Pumps.** All animal protocols were in accordance with the regulations of the Regierungspräsidium Tübingen and the local ethics committee. Following the induction of anesthesia with ketamine and xylazine the mouse is put in a prone position on a heating pad to ensure maintenance of adequate body temperature. After making an approx. 1.5 cm midline incision on the thoracic vertebrae, a pouch is created by blunt dissection to the caudal area for later implantation of the pump. The animal is put in a supine position and the hair on the right neck area is removed and desinfection of the area is done. A 1 cm incision is done and the right jugular vein is exposed by blunt dissection. The animal is now put in a lateral position and, using a forceps, a tunnel from the dorsal pocket to neck incision is done and the pump tube is put through. The animal is returned in supine position and three surgical sutures passed the jugular vein, while the cranial suture is used to completely occlude blood flow. The other two are used to hold the catheter in place after cannulation. A secure knot is tied around the vein using the cranial sutures while the other two are used making only a loose knot. Using a microscissor a small cut into the vein is done and the catheter is placed into it. By pulling the loose ends of the caudal sutures the catheter is secured in the vein. The suture tails are trimmed and the skin is closed with 5-0 non-absorbable suture **(Suppl. Fig. 3).** The administration of LEs was started after surgery. The flow rate of the infusion was set at 10 μl per hour with a concentration of 200 μg/μl (approx. 2 mg/g body weight per day).

**Intravital Microscopy of Cremaster Microvasculature.** ZyA induced peritonitis was implemented as described above. Following the induction of anesthesia, a catheter was placed in the left jugular vein for the administration of drugs and antibodies. Rhodamine-6G (Sigma-Aldrich) (100 µL, 0.05%) was injected i.v. to stain circulating leukocytes. The cremaster microcirculation was observed *in vivo* using a Nikon Eclipse Ci-L microscope (Nikon, Düsseldorf, Germany) equipped with a 20× objective lens (Objective CFI Super Plan Fluor ELWD with N.A. 0.45; Nikon) and a 100 W fluorescent lamp (C-HGFIE Intensilight, Nikon). Images were captured with an Orca-R2 Hamamatsu camera (Hamamatsu Photonics, Hamamatsu, Japan) 4 h after induction of peritonitis. For image acquisition and analysis, NIS-AR elements software (Nikon) was used. Leukocyte-endothelium interactions in the microvasculature were analyzed in a standard field (SF) per 5,000 µm^2^ of vessel surface. Rolling leukocyte flux was defined as the number of rhodamine-stained leukocytes moving slower than erythrocytes over 30 sec within the SF. Leukocytes were considered adherent if remaining stationary at the vessel wall for at least 30 sec.

**Human MΦ Efferocytosis and Phagocytosis.** For the differentiation of MΦ, peripheral blood monocytes were isolated from healthy volunteers or human leukapheresis collars from the Blood Bank of Eberhard Karls University of Tübingen and cultured in RPMI 1640 medium with 10 ng/mL human recombinant GM-CSF (R&D Systems) (37 °C for 7 days). To prepare apoptotic PMNs, human PMNs obtained from peripheral blood were isolated and labeled with carboxyfluorescein diacetate (10 μM, 30 min at 37 °C; Molecular Probes) and allowed to undergo apoptosis in serum-free RPMI 1640 medium for 16-18 h. MΦ (0.1 x 10^6^ cells/well) were then incubated with Ω-3^+^ (Lipidem, B.Braun) or Ω-3^-^ (Lipofundin, B.Braun) LEs or vehicle. Apoptotic PMNs were added at a 1:3 ratio (MΦ:PMN) and incubated at 37 °C for 60 min to induce phagocytosis. In separate experiments, MΦ were incubated either with Ω-3^+^ or Ω-3^-^ LE or vehicle for 15 min at 37 °C and then incubated with ZyA particles (Molecular Probes, Darmstadt, Germany) at a 1:30 ratio (MΦ:ZyA particles) or E-coli particles at a 1:50 ratio (MΦ:E.coli) for 60 min. In a further experiment, human MΦ were incubated with 0.1, 1.0, 10, 100 and 1000 μg of baicalein or CDC and the degree of phagocyted fluorescently labeled ZyA particles was assessed. Fluorescence was determined by using a fluorescent plate reader (Tecan, Männedorf, Switzerland).

**LC-MS/MS.** Peritoneal lavage samples were spiked with 4 µL of an internal standard solution (containing PGE_4_-d4, LTB_4_-d4 15-HETE-d8 and DHA-d6 at a concentration of 50 ng/ml in methanol). The samples were transferred to a 12-ml glass vial, and 1.75 ml of methanol was added. The samples were centrifuged at 4,000 rpm for 5 min at 6 °C, and the supernatant was transferred to a fresh 12-ml glass vial. The pellet was re-extracted with 500 µl of methanol and centrifuged as described above, and the organic extracts were combined. The methanol was partially removed under a gentle stream of nitrogen at 40 °C for 30 min. The remaining methanolic extract (approximately 1.5 ml) was diluted with 8 ml of water, and 20 µl of 6 M HCl was added. The prepared samples were cleaned via solid phase extraction (SPE) (SepPak C18 200 mg, Waters, MA, USA). The samples were loaded onto preconditioned SPE cartridges (2 ml methanol, followed by 2 ml water), the cartridges were washed with 3 ml of water followed by 3 ml of *n*-hexane, and then the samples were eluted with 3 ml of methylformate. The eluate was dried under a gentle stream of nitrogen, reconstituted in 200 µL of 40% methanol, and injected.

LC-MS/MS analysis was performed as described below. Briefly, a QTrap 6500 mass spectrometer operating in negative ESI mode (Sciex, Nieuwerkerk aan den Ijssel, The Netherlands) was coupled to an LC system employing two LC-30AD pumps, a SIL-30AC autosampler, and a CTO-20AC column oven (Shimadzu, ’s-Hertogenbosch, The Netherlands). A 1.7 µm Kinetex C_18_ 50 × 2.1 mm column protected with a C8 precolumn (Phenomenex, Utrecht, The Netherlands) was used, and the column was maintained at 50 °C. A binary gradient of water (A) and MeOH (B) containing 0.01% acetic acid was generated as follows: 0 min 30% B, held for 1 min, then ramped to 45% B at 1.1 min, 53.5% B at 2 min, 55.5% B at 4 min, 90% B at 7 min, and 100% B at 7.1 min, and held for 1.9 min. The injection volume was 40 µl, and the flow rate was 400 µl/min. The MS was operated as previously described^1^. For analyte identification, the mass transition used for each analyte was combined with its relative retention time (RRT). The calibration lines constructed with standard material for each analyte were used for quantification, and only peaks with a signal to noise (S/N) ratio > 10 were quantified.

**Suppl. Figure Legends**

**Suppl. Figure 1: Overview of the Ω-3^+^ and Ω-3^-^ LEs induced pro-resolving lipid mediator biosynthesis at 4 h and 12 h.** WT mice were exposed to Ω-3^+^ or Ω-3^-^ LE or vehicle for 24 h before injecting ZyA and then collecting peritoneal lavages at 4 h, 12 h, 24 h and 48 h. LC-MS/MS based profiling was performed. Levels of bioactive lipid mediators and precursors derived from the AA, DHA and EPA pathways. The results represent two to three independent experiments with n=6-8 mice/group. All results are reported as ng/ml. For substances where no calibration line was constructed, the values represent area ratios to the specific internal standard.

**Suppl. Figure 2: FACS gating strategy for leukocyte differentiation and efferocytosis.** Peritoneal lavages were prepared as described in the Material and Methods. Leukocytes were gated on FSC/SSC. Leukocyte subtypes were further classified into Ly6G^hi^, Ly6C^hi^ and Ly6C^lo^. For defining efferocytosis, the differentiation of intra- and extra-cellular PMN was assessed by using Ly6G-PerCP-Cy5.5 and Ly6G-APC antibodies. Phagocytized PMNs were Ly6G-PerCP-Cy5.5^+^ and Ly6G-APC^-^.

**Suppl. Figure 3: Implantation of infusion pumps.** The method is described in detail in the Material and Methods.

**Suppl. Figure 4: Ω-3^+^ LEs stimulate resolution of inflammation in murine polymicrobial sepsis.** WT mice were administered with Ω-3^+^ LEs, Ω-3^-^ LEs or vehicle 24 h prior exposure to CLP and lavages were collected at 4 h. **A)** Total leukocytes were enumerated by light microscopy and PMNs were characterized by flow cytometry. **B)** Classical monocytes, non-classical monocytes and efferocytosis were determined. Results represent two independent experiments and are expressed as the mean±SEM, n=6-8 per group, *P<0.05; **P<0.01; ***P<0.001, One-way ANOVA followed by Bonferroni’s multiple-comparison test.

**Suppl. Figure 5: Ω-3^+^ LEs enhance pro-resolving lipid mediator biosynthesis in murine polymicrobial sepsis.** WT mice were administered with Ω-3^+^ LEs, Ω-3^-^ LEs or vehicle 24 h prior exposure to CLP and lavages were collected at 4 h. LC-MS/MS based profiling was performed in murine peritoneal lavages. All results are reported as ng/10^6^ cells of peritoneal lavage. Results represent two independent experiments and are expressed as the mean±SEM, n=6-8 per group, *P<0.05; **P<0.01; ***P<0.001, One-way ANOVA followed by Bonferroni’s multiple-comparison test.

**Table S1: Composition of Ω-3^+^ or Ω-3^-^ LEs.**

**Table S2: Lipid mediator levels in murine peritoneal lavages following administration of Ω-3^+^, Ω-3^-^ LEs or vehicle.** LC-MS/MS based profiling was performed in peritoneal lavages of C57BL/6 mice exposed to Ω-3^+^ LE or vehicle for 24 h before inducing ZyA peritonitis for 4 h. The results represent two to three independent experiments with n=6-8 mice/group. All results are reported as ng/ml. For substances where no calibration line was constructed, the values represent area ratios to the specific internal standard.

**Suppl. Movie 1A: 24 hours after CLP:** General state after treatment with Ω-3^+^ LEs.

**Suppl. Movie 1B: 24 hours after CLP:** General state after treatment with Ω-3^-^ LEs.

**Suppl. Movie 1C: 24 hours after CLP:** General state after treatment with Ω-3^+^ (left cage) or Ω-3^-^ LEs (right cage).

**Suppl. Movie 1D: 72 hours after CLP:** General state after treatment with Ω-3^+^ LEs.

**Suppl. Movie 1E: 72 hours after CLP:** General state after treatment with Ω-3^-^ LEs.

**Suppl. Movie 1F: 72 hours after CLP:** General state after treatment with Ω-3^+^ (left cage) or Ω-3^-^ LEs (right cage).

**Suppl. Movie 2A:** This file shows a 20 sec time-lapse movie of rolling (red) and adherent leukocytes (yellow) in peritonitis mice treated with Ω-3^+^ LEs.

**Suppl. Movie 2B:** This file shows a 20 sec time-lapse movie of rolling (red) and adherent leukocytes (yellow) in peritonitis mice treated with vehicle.

**REFERENCES**

1. Heemskerk MM, Dharuri HK, van den Berg SA, Jonasdottir HS, Kloos DP, Giera M*, et al.* Prolonged niacin treatment leads to increased adipose tissue PUFA synthesis and anti-inflammatory lipid and oxylipin plasma profile. *J Lipid Res* 2014, **55**(12)**:** 2532-2540.
