# Supplementary figures and images for: Resolution of inflammation and sepsis survival are improved by dietary Ω-3 fatty acids
Source: Cell Death Differ. 2017 Oct 20;25(2):421–31. doi: 10.1038/cdd.2017.177 (PMC5762854; doi:10.1038/cdd.2017.177)

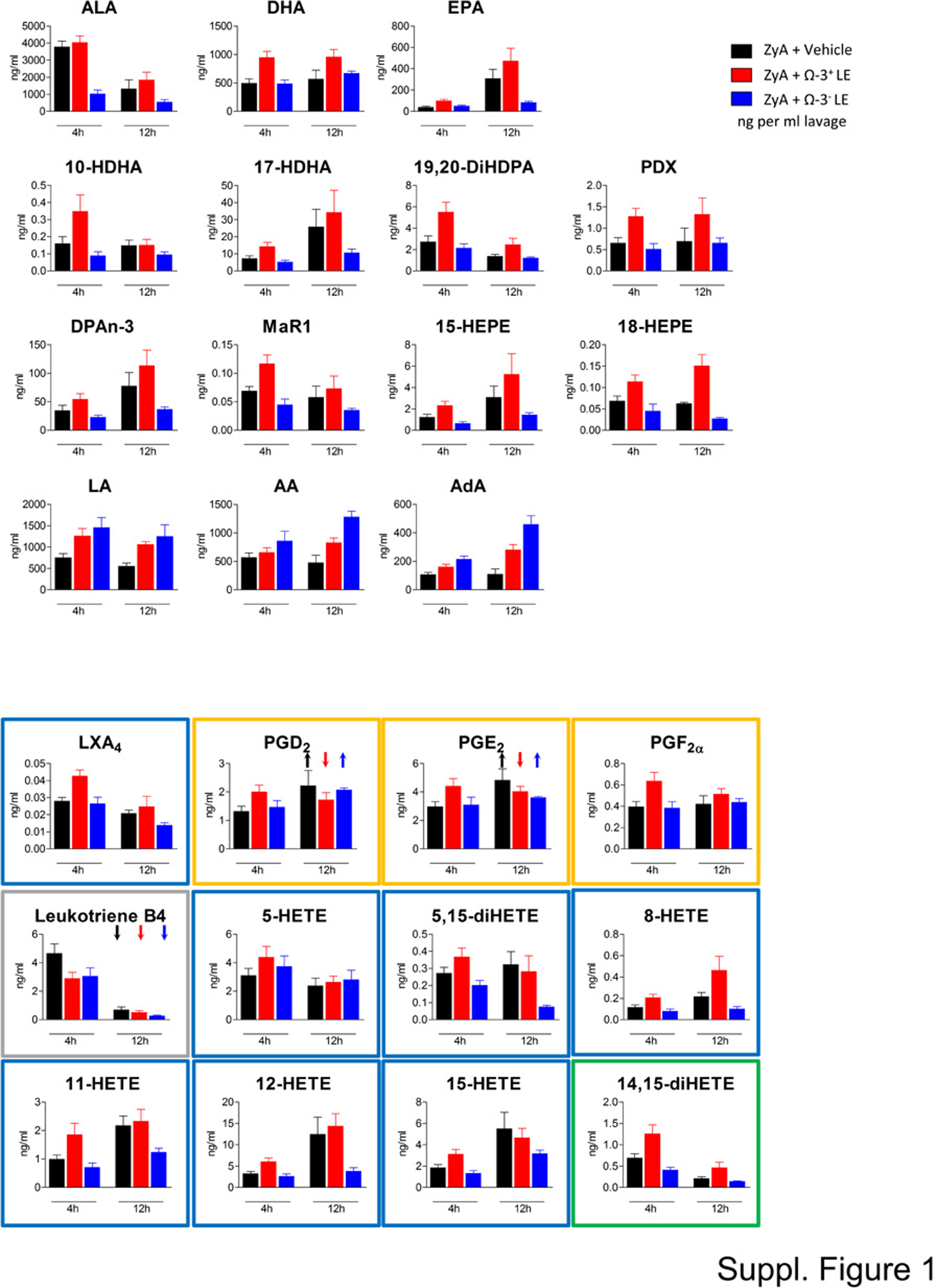

Supplement: Supplementary Figure 1 [file cdd2017177x2.tif]

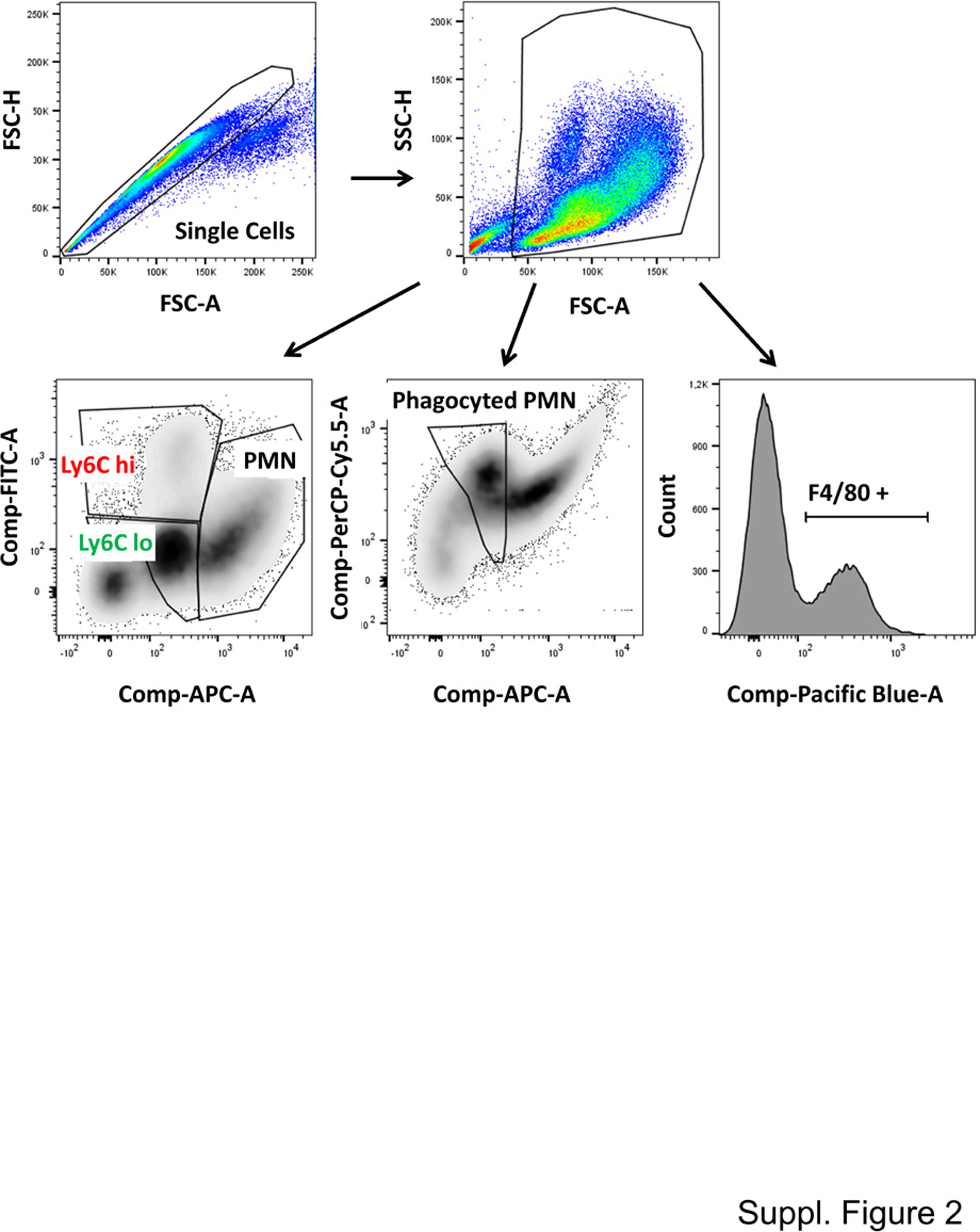

Supplement: Supplementary Figure 2 [file cdd2017177x3.tif]

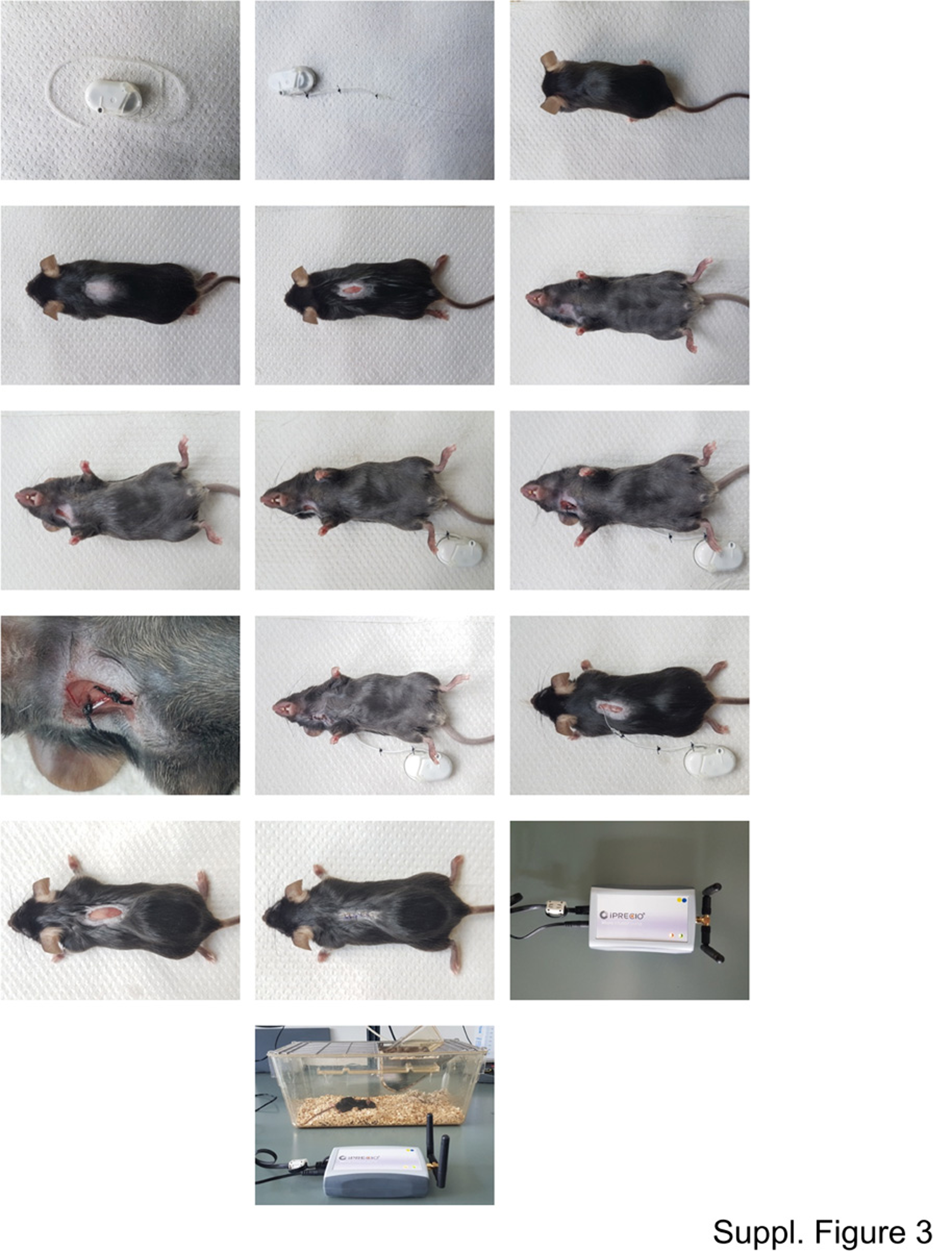

Supplement: Supplementary Figure 3 [file cdd2017177x4.tif]

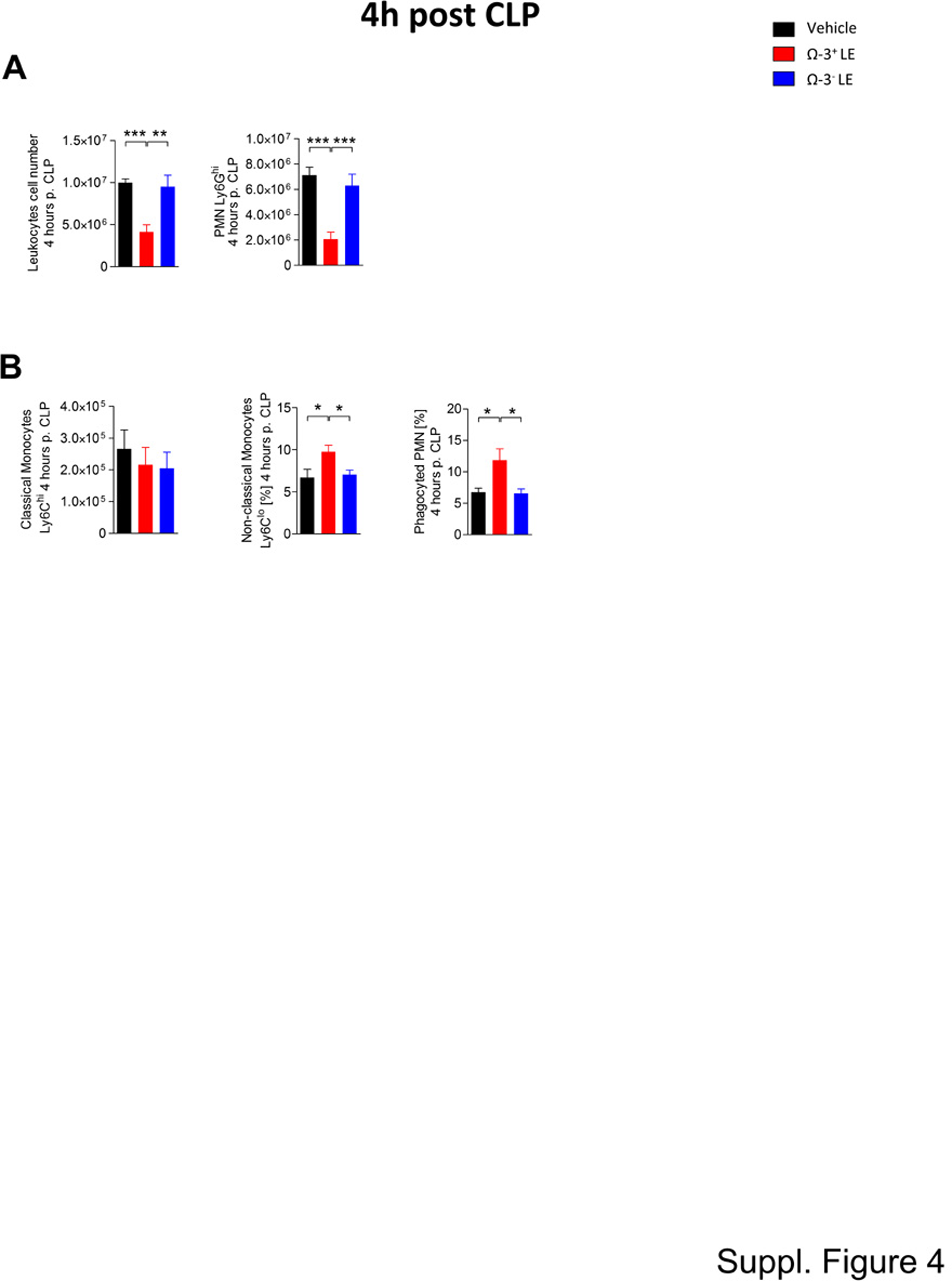

Supplement: Supplementary Figure 4 [file cdd2017177x5.tif]

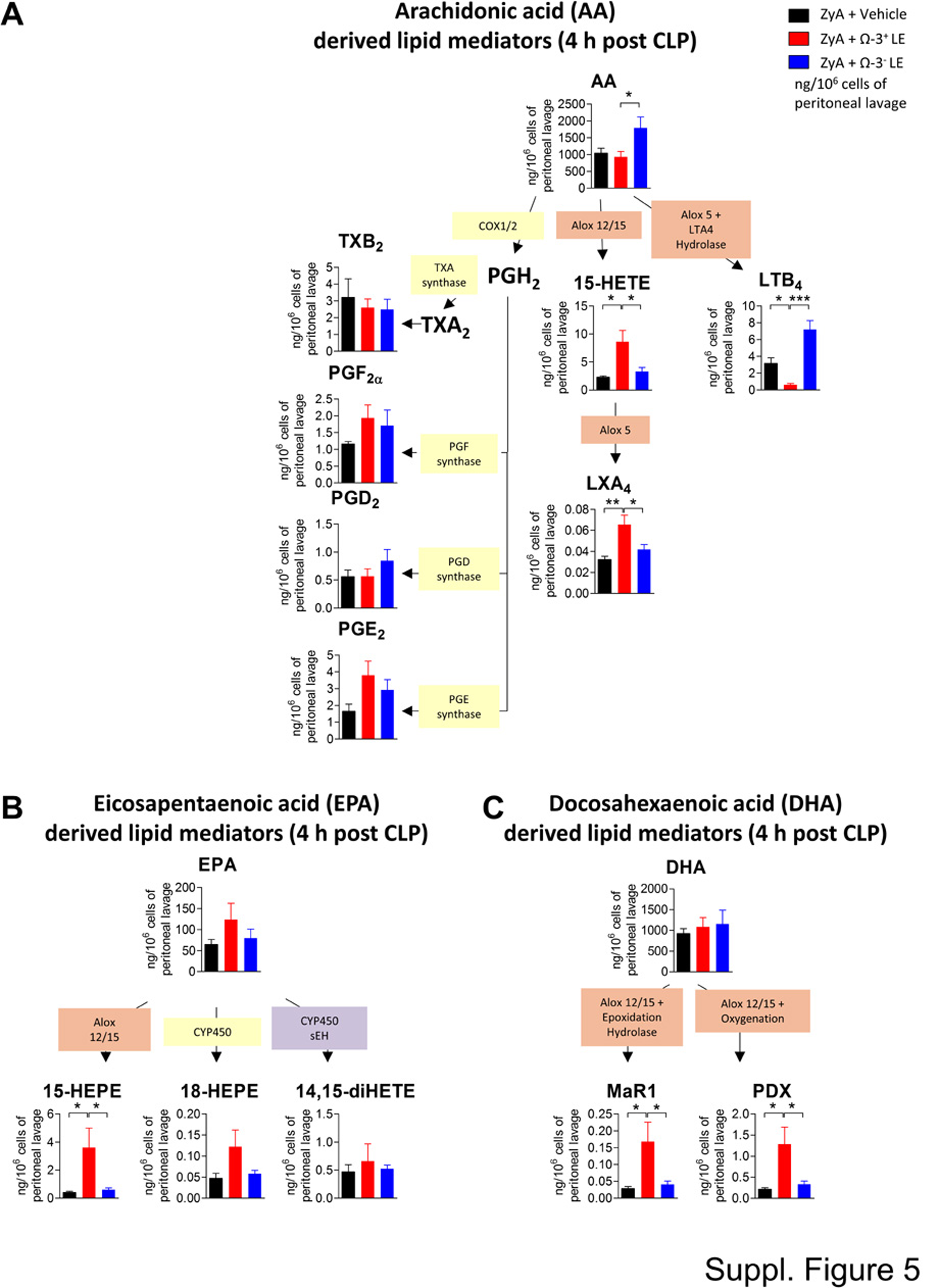

Supplement: Supplementary Figure 5 [file cdd2017177x6.tif]
