# Supplementary material for: Resolution of inflammation and sepsis survival are improved by dietary Ω-3 fatty acids
Source: Cell Death Differ. 2017 Oct 20;25(2):421–31. doi: 10.1038/cdd.2017.177 (PMC5762854; doi:10.1038/cdd.2017.177)
Supplement: Supplementary Table 1 [file cdd2017177x7.docx]

| **Lipid emulsion** | **Lipid composition** | **n-6 : n-3 ratio** | **Osmolarity**  **[mOsm/kg]** | **Energy [kJ/l (kcal/l)** |
| --- | --- | --- | --- | --- |
| Ω-3^+^ LE | **MCT:LCT:FO 50:40:10**  19-23% (21%) LA (n-6)  2-4,4% (3,2%) ALA (n-3)  4,3-8,6% (6,45%) EPA, DHA (n-3) | 2,2 : 1 | 410 | 7990  (1910) |
| Ω-3^-^ LE | **LCT:MCT 50:50**  24-29% (26,5%) LA (n-6)  2,5-5,5% (4%) ALA (n-3) | 6,6 : 1 | 380 | 8095  (1935) |

Table S1
