# Supplementary material for: Resolution of inflammation and sepsis survival are improved by dietary Ω-3 fatty acids
Source: Cell Death Differ. 2017 Oct 20;25(2):421–31. doi: 10.1038/cdd.2017.177 (PMC5762854; doi:10.1038/cdd.2017.177)
Supplement: Supplementary Table 2 [file cdd2017177x8.docx]

| **Metabolome** | **Vehicle** | | **Ω-3^+^ LE** | | **Ω-3^-^ LE** | | **p-value** | |
| --- | --- | --- | --- | --- | --- | --- | --- | --- |
|  | **Mean** | **SEM** | **Mean** | **SEM** | **Mean** | **SEM** | **Vehicle / Ω-3^+^ LE** | **Ω-3^+^ LE / Ω-3^-^ LE** |
|  | **ng/ml** | | | | | |  | |
| **AA** | 568.50000 | 77.42000 | 657.40000 | 81.47000 | 645.10000 | 80.48000 | 0.4719 | 0.9357 |
| **DHA** | 496.00000 | 72.42000 | 949.30000 | 104.70000 | 487.30000 | 60.67000 | 0.0034 ** | 0.0344 * |
| **EPA** | 40.14000 | 7.58400 | 99.01000 | 14.34000 | 49.78000 | 6.86700 | 0.0032 ** | 0.0431 * |
| **LA** | 754.00000 | 93.17000 | 1265.00000 | 168.70000 | 1458.00000 | 232.50000 | 0.0491 * | 0.5097 |
| **ALA** | 3780.00000 | 338.90000 | 4050.00000 | 361.50000 | 1032.00000 | 212.30000 | 0.6009 | < 0.0001 *** |
| **AdA** | 107.50000 | 14.17000 | 161.50000 | 17.90000 | 214.40000 | 22.49000 | 0.0349 * | 0.1236 |
| **LXA_4_** | 0.02815 | 0.00197 | 0.04267 | 0.00356 | 0.02657 | 0.00362 | 0.0038 ** | 0.0251 * |
| **5-HETE** | 3.10800 | 0.49910 | 4.40600 | 0.74210 | 3.75700 | 0.71940 | 0.2072 | 0.6308 |
| **8-HETE** | 0.11600 | 0.02089 | 0.20790 | 0.03158 | 0.07954 | 0.02006 | 0.0423 * | 0.0372 * |
| **11-HETE** | 0.99900 | 0.13880 | 1.63600 | 0.36210 | 0.70730 | 0.15140 | 0.1894 | 0.1547 |
| **12-HETE** | 3.27700 | 0.44140 | 6.04000 | 0.85570 | 2.62000 | 0.53670 | 0.0295 * | 0.0311 * |
| **15-HETE** | 1.86300 | 0.28640 | 3.14600 | 0.42820 | 1.33000 | 0.24470 | 0.0342 * | 0.0412 * |
| **15-HEPE** | 1.22700 | 0.26150 | 2.33400 | 0.38780 | 0.64760 | 0.14430 | 0.0445 * | 0.0395 * |
| **18-HEPE** | 0.06896 | 0.01116 | 0.11420 | 0.01560 | 0.04549 | 0.01574 | 0.0373 * | 0.0184 * |
| **Leukotriene B_4_** | 4.65900 | 0.66790 | 2.91200 | 0.41970 | 3.05900 | 0.58980 | 0.0272 * | 0.8572 |
| **17-HDHA** | 7.29600 | 1.54600 | 14.32000 | 2.35100 | 5.21800 | 0.96500 | 0.0402 * | 0.0487 * |
| **14,15-diHETE** | 0.69360 | 0.09517 | 1.26300 | 0.20290 | 0.41280 | 0.05571 | 0.0392 * | 0.0336 * |
| **19,20-DiHDPA** | 2.73400 | 0.53720 | 5.53300 | 0.90040 | 2.14500 | 0.40340 | 0.0261 * | 0.0663 |
| **TXB_2_** | 3.99300 | 0.69220 | 4.06600 | 0.76170 | 3.52600 | 0.77080 | 0.9448 | 0.6398 |
| **6-trans-LTB_4_** | 1.66400 | 0.22070 | 1.90900 | 0.47850 | 0.75240 | 0.09284 | 0.6894 | 0.1899 |
| **PGD_2_** | 1.32400 | 0.17740 | 2.01000 | 0.23050 | 1.46900 | 0.23550 | 0.0422 * | 0.2413 |
| **PDX** | 0.65270 | 0.12500 | 1.28000 | 0.18690 | 0.51220 | 0.12830 | 0.0227 * | 0.0395 * |
| **MaR1** | 0.06926 | 0.00737 | 0.11720 | 0.01481 | 0.04479 | 0.01001 | 0.0432 * | 0.0186 * |
| **LTE4** | 0.10220 | 0.01524 | 0.08831 | 0.01708 | 0.10670 | 0.04872 | 0.5658 | 0.6531 |
| **8S,15S-diHETE** | 0.21600 | 0.06584 | 0.21650 | 0.03757 | 0.14430 | 0.04138 | 0.9941 | 0.2813 |
| **7,17-DiHDPA** | 0.02653 | 0.00000 | 0.08540 | 0.04081 | --- | --- | --- | --- |
| **6t,12-epi-LTB_4_** | 2.39500 | 0.36440 | 2.45800 | 0.52150 | 0.95470 | 0.13350 | 0.9294 | 0.1210 |
| **PGF_2α_** | 0.39640 | 0.04790 | 0.63550 | 0.08213 | 0.38250 | 0.06023 | 0.0357 * | 0.1316 |
| **PGE_2_** | 2.97800 | 0.34200 | 4.40900 | 0.53940 | 3.10500 | 0.51800 | 0.0447 * | 0.2371 |
| **15-keto-PGE_2_** | 0.07596 | 0.00789 | 0.11550 | 0.01502 | 0.07155 | 0.01280 | 0.0490 * | 0.1528 |
| **13,14-dihydro-15 keto-PGF_2α_** | 0.22480 | 0.02564 | 0.36750 | 0.06047 | 0.26110 | 0.04740 | 0.0894 | 0.2075 |
| **8-iso-PGE_2_** | 0.39790 | 0.07676 | 0.52000 | 0.07690 | --- | --- | 0.2873 | --- |
| **8-iso-PGF_2α_** | 0.06061 | 0.00783 | 0.13640 | 0.04861 | --- | --- | 0.1828 | --- |
| **DPA n-3** | 34.72000 | 9.14100 | 54.78000 | 9.74600 | 22.91000 | 3.33100 | 0.1629 | 0.0848 |
| **PGJ_2_** | 397.10000 | 140.40000 | 375.30000 | 128.60000 | 0.21750 | 0.03916 | 0.9115 | 0.2590 |
| **5,15-diHETE** | 0.27300 | 0.03374 | 0.36880 | 0.04985 | 0.20230 | 0.02666 | 0.1576 | 0.0379 * |
| **10-HDHA** | 0.16070 | 0.03855 | 0.34960 | 0.09473 | 0.08986 | 0.02208 | 0.1293 | 0.0435 * |
| **7-HDHA** | 0.21430 | 0.03757 | 0.24630 | 0.04187 | 0.22150 | 0.04293 | 0.5942 | 0.7417 |
| **14,(15)EET** | 0.22360 | 0.04497 | 0.48180 | 0.09573 | --- | --- | 0.0255 * | --- |

Table S2
